# Effect of problem-based learning approach program on meta-cognitive thinking skills and cooperative learning attitude among nursing students: quasi experimental study

**DOI:** 10.1186/s12912-025-03485-z

**Published:** 2025-07-10

**Authors:** Shymaa Abdelhafez Mohamed, Sanaa Abd Elazim Ibrahim, Marwa Mohamed Abdelaalem, Noura Elgharib Mohamed Eldiasty

**Affiliations:** Nursing Administration Department, Faculty of Nursing, Port Said University, Port Said, Egypt

**Keywords:** Cognitive process, Cooperative learning attitude, Critical thinking, Nursing education, Metacognitive thinking skills, Nursing students, Problem-based learning, Student-centre learning, Team work

## Abstract

**Aim:**

This study aimed to evaluate the effect of problem-based learning approach program on meta -cognitive thinking skills and cooperative learning attitude among nursing students.

**Background:**

Nursing students encounter numerous challenges and obstacles that can hinder their academic performance. The problem-based learning approach is designed to enhance metacognitive thinking skills and cooperative learning attitude. These elements lead to master, directed and adaptable to the problems occurring within the academic and practical environment.

**Methods:**

The intervention commenced in the period started from 28 September 2023 to 28 October 2023 through workshop with a preparatory phase at the beginning of the study, A quasi-experimental design was employed at the Faculty of Nursing in Port Said, Egypt. The study involved 207 nursing students enrolled in the first and incoming second academic levels for the 2023–2024 academic year. Data were collected by the Problem-Based Learning Approach Knowledge Questionnaire, the Metacognition Self-Assessment Scale, and the Students’ Cooperative Learning Attitude Questionnaire. The data analysis procedures which the study employed version 27.0 of the Statistical Package for Social Sciences (SPSS) for data analysis using Shapiro–Wilk test and single-factor (time) repeated measures analysis of variance (ANOVA) with Greenhouse–Geisser correction.

**Results:**

The findings illustrated significant improvements in participants’ meta-cognitive thinking skills over time (F = 904.39; *p* < 0.001). Additionally, there was a notable significant change over time in participants’ cooperative learning attitudes (F = 822.74; *p* < 0.001).

**Conclusion:**

The problem-based learning program effectively enabled nursing students to develop mastery in meta-cognitive thinking skills and improved cooperative learning attitudes.

**Implications:**

The study offers several important implications for nursing education practice and policy. These skills are critical for the practice of nursing. Educational policymakers and curriculum developers should consider integrating problem-based learning strategies more comprehensively within nursing programs to foster essential skills in critical thinking, problem-solving, and collaboration.

**Trial registration number:**

The study protocol was approved by the Research Ethics Committee of the Faculty of Nursing, Port Said University (code number: NUR 7/5/2023 (25)).

**Supplementary Information:**

The online version contains supplementary material available at 10.1186/s12912-025-03485-z.

## Introduction

In contemporary educational paradigms, learning is a pivotal concern across all forms of education, with the shift towards virtual environments catalyzing a transition from fact-based to inquiry-based learning methods [1]. Consequently, problem-based learning (PBL) has become essential as a method of self-directed learning [2], characterized by an approach where learning results from efforts directed towards understanding or resolving a problem [3].

PBL is a crucial educational strategy in nursing education, fostering critical analytical skills and problem-solving capabilities, enhancing student motivation and engagement, and developing essential interpersonal and teamwork skills through the use of real-world scenarios and cases [4]. The fundamental techniques employed in PBL include group discussions, individual research and reflection, role-playing, case simulations, self-evaluation, and the utilization of multimedia tools and technologies [5].

Educational institutions play an essential role in the success of PBL programs in nursing education, responsible for their development, funding, evaluation, and providing necessary training and support to students, tutors, and staff [6]. Meeting these responsibilities is crucial to ensuring that PBL programs are effective, relevant, and contribute to meaningful student learning and development [6].

Metacognitive thinking, a complex and multifaceted cognitive process, involves self-awareness, self-monitoring, and self-regulation. Effective instruction and practice of meta-cognitive strategies can significantly improve students’ academic performance and their ability to handle complex learning tasks [7]. This type of thinking includes an awareness of one’s cognitive processes and the proactive ability to control and regulate one’s own learning experience [8]. Winne added that meta-cognitive thinking is crucial across various educational settings and is essential for lifelong learning [9].

Employing meta-cognitive strategies enhances awareness, aids in progress monitoring, and facilitates the adaptation of learning strategies, thereby fostering academic and personal achievements [10]. Both cognitive and meta-cognitive processes are essential for students to become adept, self-regulated learners. At an organizational level, promoting meta-cognitive practices can enhance the development of learning strategies and support learners’ educational and developmental progress. Recognizing and addressing organizational factors in higher education can lead to environments that promote effective learning strategies and meta-cognitive awareness, thereby enhancing academic success [10, 11].

Therefore Pedone et al. [12] asserted that, the problem based learning process must be improved to improve its impact on metacognitive thinking skills as in a practical environment, problem based learning acts as catalyst for simultaneous development of cognitive thinking skills and metacognitive awareness. it’s not only helps learner solve problems more effectively but also understand and control how they learn preparing them to be adaptive, reflective professionals capable of navigating complex, real – world challenges.

Prior research integrating various forms of cooperative learning represents a crucial and effective pedagogical approach that enhances student learning, fosters social and emotional growth, and prepares students for success in diverse contexts [13, 14]. This strategy incorporates elements that collectively cultivate a supportive and inclusive educational environment, allowing students to develop their social, emotional, and cognitive abilities [15]. Moreover, Cooperative learning’s underlying theory emphasizes its effectiveness in promoting social cohesion, facilitating active and meaningful learning experiences, boosting motivation, and reducing cognitive load [16].

Evaluating students’ attitudes towards cooperative learning, particularly during discussions and debriefing sessions, demands a multifaceted approach. Techniques such as direct observation, self-assessment, peer feedback, group debriefing, attitude measurement scales, and performance evaluations are invaluable [17]. In addition to, by assessing students’ dispositions towards cooperative learning, educators can pinpoint areas requiring enhancement and modify their instructional strategies to better meet the needs of their students, thereby optimizing the learning experience and outcomes [18].

So, the problem based learning process must be improved to improve its impact on cooperative learning attitudes as problem based learning and cooperative learning attitudes are synergistic in practical environment. PBL fostering the need for collaboration, while a cooperative attitude enhances the success and depth of problem solving. Together, they prepare learners for real – world challenges by mimicking professional collaboration and nurturing lifelong learning skills [16].

The study posits the following research questions and hypotheses to be answered and tested after students in the first and second levels participate in a problem-based learning program:

### Research questions

Is the program will be enhanced students’ knowledge of the problem-based learning approach?

Are students’ meta-cognitive thinking skills will be enhancement?

Are students’ cooperative learning attitudes will be improved?

### Research hypothesis

#### Hypothesis 1

There will be an enhancement in students’ knowledge of the problem-based learning approach.

#### Hypothesis 2

Students will exhibit enhanced meta-cognitive thinking skills.

#### Hypothesis 3

There will be an improvement in students’ cooperative learning attitudes.

## Subjects and methods

### Study design

A quasi-experimental design was implemented in this study.

### Participants and setting

The research was conducted at the Faculty of Nursing at Port Said University, which employs a problem-based learning approach as its core educational system. The study targeted all nursing students registered in the first academic level and newcomers in the second level for the 2023–2024 academic year. We have calculated the post hoc (observed) power for our main analyses given the achieved sample size (*N* = 207) and the observed effect sizes. Using G*Power (Version 3.1.9.7) for a repeated measures ANOVA (within factors), with *N* = 207, 3 measurement points, and an alpha of 0.05, the achieved power for our main study outcomes is > 0.99.

### Inclusion criteria

Nursing students who are newly admitted to the faculty are included in the analysis(first level of students and second level of students who enrolled the faculty from from nursing institution).

### Exclusion criteria

Nursing students who in the second year and successfully completed the first year at the faculty, as well as those who were enrolled in another program within the six months before data collection, are excluded.

In total, 361 students were initially considered for the study. For the first level, 177 students were invited; 18 participated in a pilot study, 19 declined to participate in the post-test, and 20 did not complete the follow-up test, leaving 120 students who fully participated in the study. In the second level, 184 students were invited; 65 did not meet the inclusion criteria, 12 were involved in the pilot testing, 10 did not complete the post-test, and 10 declined to participate in the follow-up test, resulting in 87 students who completed the study. In total, 207 students have already participated in the study.

### Intervention

The problem-based learning (PBL) approach program was developed following a thorough review of relevant literature. The intervention aimed to enhance students’ knowledge of PBL, which subsequently improved their PBL outcomes.

Pre-intervention Preparation: Before the intervention program, potential students from different levels were interviewed to understand their perspectives on several aspects of their educational experience. These included their definitions of good performance, obstacles hindering such performance, supports that facilitated high outcomes in PBL, tasks they found challenging, and reasons for not overcoming these challenges. The insights gathered from these interviews were used to tailor the intervention and customize examples for the intervention.

Implementation of the Intervention: The program was introduced by corresponding author and it was commenced in the period started from 28 September 2023 to 28 October 2023 through workshop with a preparatory phase at the beginning of the study, consisting of a three-day interactive sessions per two week for first-level students, with approximately 20 students participating each day. The second-level students were divided into groups for a four-day interactive sessions, with about 22 students each day, held over one week. Each interactive session included three sessions, lasting 60–90 min, with a 30-minute rest between sessions. Session 1: The researcher presented the theoretical background of PBL. Session 2: The PBL process was initiated, and participants were asked to form small groups to engage in problem-solving tasks. Session 3: An open discussion was facilitated, focusing on the PBL approach.

At the conclusion of the discussions, participants were expected to have a clear understanding of the benefits of PBL, its fundamental principles, implementation processes, and strategies to overcome common pitfalls and obstacles. Each participant received a booklet containing the teaching materials developed from an intensive literature review and tailored to the students’ needs identified through the initial interviews.

Post-Workshop Engagement, students engaged in brainstorming and debriefing sessions for four weeks, guided by the PBL approach booklet developed by the researcher. During this period, students were encouraged to undertake activities related to the PBL approach and to address challenges in the first, second, third, and fourth weeks respectively.

To facilitate continuous engagement and support, a WhatsApp group was created. This platform served to encourage participation, guide discussions, share experiences, and send reminders twice weekly to engage in the prescribed activities. Reflection and Evaluation After the four-week intervention program, a reflection session was conducted where students evaluated their achievements, discussed obstacles and solutions, and planned future strategies to enhance their PBL outcomes. At the end of this session, the researcher expressed appreciation to all participating students.

### Measures

The study utilized three instruments to collect data, including the Problem-Based Learning Approach Knowledge Questionnaire, the Metacognition Self-Assessment Scale, and the Cooperative Learning Attitude Questionnaire. The tools which were originally developed in English and translated into Arabic and was evaluated by committee approach. The committee, composed of seven Egyptian nursing academic professors fluent in English, translated the questionnaires and scale independently and in parallel. The members unanimously approved the final versions of each instrument. All questionnaires and scales were self-administered by participants at three different time points during the study.

*Problem-Based Learning Approach Knowledge Questionnaire*: This questionnaire was developed by the researcher, drawing from a literature review [5, 19–23]. It aims to assess the knowledge of the problem-based learning approach among first and second-level nursing students. The questionnaire comprises 50 items divided into two categories: 30 true/false items and 20 multiple-choice items. Scoring is based on a cutoff value of 60%, as per the scoring system [24], categorizing knowledge into inadequate (< 60%) and adequate (≥ 60%). This threshold was selected as it aligns with the standard pass mark for foundational knowledge assessments within the Faculty of Nursing at Port Said University, representing a generally accepted level of basic understanding.

*Metacognition Self-Assessment Scale (MSAS)*: Developed by Pedone et al. [12] and later adopted from Faustino et al. [25], this scale measures multifunctional metacognitive skills among nursing students. It consists of 18 items across four dimensions: Understanding One’s Own Mind (5 items), Decentering and Differentiation (6 items), Mastery (4 items), and Understanding Other’s Minds (3 items). Responses are scored on a 5-point Likert scale, ranging from 1 (never) to 5 (almost always), with higher scores indicating greater meta-cognitive skills. In our study, its reliability across the three time points was acceptable (α = 0.82–0.90).

#### Students’ cooperative learning attitude questionnaire:

Adopted from Rivas et al. [8], this questionnaire explores nursing students’ attitudes towards the cooperative learning method. It includes 13 items in both open-ended and close-ended formats. Responses are scored on a 5-point Likert scale, where 1 represents “disagree” and 5 represents “always agree.” Higher scores indicate a more favorable attitude towards cooperative learning among nursing students. In our study, its reliability across the three time points was acceptable (α = 0.80–0.93).

#### Demographic information form:

This form collects personal and academic data from nursing students, such as gender, academic levels, pr-educational qualifications, pr-enrollment information about the faculty’s learning system, and initial awareness of PBL and its benefits at the start of the study.

### Data collection and procedure

Data collection for this study was conducted longitudinally from the beginning of September 2023 to January 2024. It consisted of a three-wave measurement process: T0 (Baseline Measurement): Collected one week before the intervention, T1 (Second Measurement): Collected immediately after the intervention, T2 (Third Measurement): Collected three months after the intervention.

Prior to data collection, necessary permissions were obtained from the Dean and Vice Dean for Education and Student Affairs. Participants were recruited through scheduled introductory lectures for each level, where the study’s purposes and procedures were thoroughly explained to all potential participants.

Nursing students who met the inclusion criteria and agreed to participate were required to sign an informed consent form. Following the baseline measurement (T0), three workshops per day were conducted, accommodating up to 18 students in the first level and 27 students in the second level per workshop. The workshops were led by the first author in the lecture amphitheater. To ensure participant retention, the program was scheduled at times convenient for the participants. Multiple communication methods—including personal phone contact, a WhatsApp group, and email—were employed to remind participants to attend the sessions and engage in subsequent brainstorming and debriefing sessions.

### Validity

The intervention program and data collection instruments were validated by seven experts in the related field of study, ensuring their appropriateness and accuracy for the targeted educational context.

### Tool reliability

Reliability of the tools was checked by testing its internal consistency using a Cronbach’s Alpha test.

### Pilot study

A pilot study was conducted to evaluate the clarity, feasibility, validity, and reliability of the study measures, the time required for data collection, and the quality and clarity of the intervention materials. This pilot involved 30 students (18 from the first level and 12 from the second level) who met the same inclusion and exclusion criteria but were not included in the main study sample. Results from the pilot study indicated that the study measures and intervention materials were understandable and required no modifications.

### Data analysis

The study employed version 27.0 of the Statistical Package for Social Sciences (SPSS) for data analysis. Normal distribution of the data was assessed using the Shapiro–Wilk test. Demographic characteristics were evaluated using counts and percentages for categorical variables, and mean and range for continuous variables.

Single-factor (time) repeated measures analysis of variance (ANOVA) was used to evaluate the changes in pr-training, post-training, and follow-up training scores for the study outcomes (i.e., problem-based learning knowledge, meta-cognitive thinking skills and cooperative learning attitude) with Greenhouse–Geisser correction. In cases where the repeated measures ANOVA yielded significant changes, pairwise comparisons using Bonferroni correction were performed for post-hoc tests.

The effect size was determined using the partial eta-squared ($\:{{\upeta\:}}_{p}^{2}$) and classified as having small (0.01–0.06), medium (0.06–0.14), or large (≥ 0.14) effects [26] criteria. The study adopted a significance level of 0.05 (two-tailed).

## Results

The demographic characteristics of the participating nursing students are summarized in Table 1. The sample comprised 207 participants, of which 60.4% were female. The participants’ ages ranged from 17 to 22 years, with a mean age of 18.88 years (SD = 1.06). The majority of participating nursing students (58.0%) were in their first level of study, and 59.9% had completed secondary school education. Only 24.6% of participants were aware of the college’s learning system prior to enrollment. However, a significant majority (88.9%) reported being made aware of the college education system at the beginning of their studies. Among those who were aware, 89.7% indicated that they benefited from the awareness and guidance provided, while 10.3% did not find it beneficial.

**Table 1:** Demographic characteristics (*N* = 207)

Characteristic	Category	no	Percent	Mean (SD)	Range
Gender	Male	82	39.6		
	Female	125	60.4		
Age (years)	≤ 20	201	97.1	18.88 (1.06)	17–22
	> 20	6	2.9		
Academic level	First level	120	58.0		
	Second level	87	42.0		
Previous education	Secondary school	124	59.9		
	Technical	83	40.1		
Did you know about the college’s learning system before joining it?	Yes	51	24.6		
	No	156	75.4		
Were you made aware of the college education system at the beginning of your studies?	Yes	184	88.9		
	No	23	11.1		
If yes: Did you benefit from this awareness and guidance?	Yes	165	89.7		
	No	19	10.3		

SD, standard deviation

According to Table 2, there was a significant effect over time on participants’ problem-based learning knowledge (F = 2880.25; *p* < 0.001), indicating that the intervention was highly effective in enhancing participants’ knowledge of problem-based learning across the study phases, with a large effect size ($\:{{\upeta\:}}_{p}^{2}$ = 0.93). The Bonferroni pairwise comparisons showed a significant increase in participants’ knowledge immediately after training completion (M (SD) = 41.13 (3.04)) compared with pr-training (M (SD) = 22.89 (3.78)), with a p-value < 0.001. This improvement was maintained three months after the program’s completion (M (SD) = 35.43 (3.60)), also with a p-value < 0.001. The results also showed a decline in participants’ knowledge mean scores from the post-training to follow-up stage, with a mean difference of 5.70 points. Despite this reduction, the follow-up mean score still demonstrated a significant improvement from the pr-intervention stage, underscoring the sustained positive impact of the intervention on participants’ problem-based learning knowledge.

**Table 2:** Changes in the mean score of participant problem-based learning knowledge over time (*N* = 207)

Variable	Pre	post	3 months Follow-up	F (*p*)	Pairwise comparison (*p*)		$\:{{\upeta\:}}_{p}^{2}$
	Mean ± SD (LB/UB)	Mean ± SD (LB/UB)	Mean ± SD (LB/UB)		Pre-post	Pre-follow	
Problem-based learning knowledge	22.89 ± 3.78 (22.37/23.41)	41.13 (3.04) )40.70/41.54)	35.43 (3.60) (34.93/35.92)	2880.25 ( < 0.001)	< 0.001	< 0.001	0.93

Abbreviations: F = one factor (time) repeated measure ANOVA; LB = Lower Bound; UB = Upper Bound

Table 3 illustrates significant improvements in participants’ meta-cognitive thinking skills over time (F = 904.39; *p* < 0.001). The total meta-cognitive thinking skills showed an increase from pr-training (M (SD) = 58.67 (9.93)) to post-training (M (SD) = 69.53 (7.33)), with a large effect size ($\:{{\upeta\:}}_{p}^{2}\:$= 0.81) and a significant p-value  < 0.001. This improvement was maintained three months after the training, as evidenced by the follow-up mean score (M (SD) = 65.67 (7.96)), despite a slight decrease of 3.86 points compared to the post-training stage. Among the individual dimensions of meta-cognitive thinking skills, “Understanding one’s own mind” improved significantly from pr-training (M (SD) = 16.22 (3.43)) to post-training (M (SD) = 18.39 (2.87)), with the improvement maintained at follow-up (M (SD) = 17.64 (3.04)), showing an effect size of $\:{{\upeta\:}}_{p}^{2}$ = 0.56. Similarly, “Decentering and differentiation” demonstrated significant growth from pr-training (M (SD) = 20.59 (3.82)) to post-training (M (SD) = 23.97 (2.73)), which was sustained at follow-up (M (SD) = 22.66 (3.03)) with an effect size of $\:{{\upeta\:}}_{p}^{2}$ = 0.69. The “Mastery” dimension also showed notable improvement from pr-training (M (SD) = 12.34 (2.84)) to post-training (M (SD) = 15.53 (2.19)), with a follow-up score of (M (SD) = 14.13 (2.38)), with a large effect size of $\:{{\upeta\:}}_{p}^{2}$= 0.67. Lastly, “Understanding others’ minds and starting activities” increased significantly from pr-training (M (SD) = 9.53 (2.30)) to post-training (M (SD) = 11.65 (1.99)), with the follow-up score (M (SD) = 11.24 (1.94)) also showing significant retention, with an effect size of $\:{{\upeta\:}}_{p}^{2}$= 0.56. These findings indicate that the training program was highly effective and lead to in enhancing participants’ meta-cognitive thinking skills across all dimensions, with may be sustain improvements over time.

**Table 3:** Changes in the mean score of participants meta-cognitive thinking skills over time (*N* = 207)

Variable	Pre	post	3 months Follow-up	F (*p*)	Pairwise comparison (*p*)		$\:{{\upeta\:}}_{p}^{2}$
	Mean ± SD (LB/UB)	Mean ± SD (LB/UB)	Mean ± SD (LB/UB)		Pre-post	Pre-follow	
Understanding one’s own mind	16.22 ± 3.43 (15.74/16.68)	18.39 ± 2.87 (17.99/18.78)	17.64 ± 3.04 (17.22/18.06)	263.71 ( < 0.001)	< 0.001	< 0.001	0.56
Decentering and differentiation	20.59 ± 3.82 (20.07/21.11)	23.97 ± 2.73 (23.59/24.34)	22.66 ± 3.03 (22.24/23.07)	390.36 ( < 0.001)	< 0.001	< 0.001	0.69
Mastery	12.34 ± 2.84 (11.94/12.73)	15.53 ± 2.19 (15.22/15.87)	14.13 ± 2.38 (13.80/14.46)	416.50 ( < 0.001)	< 0.001	< 0.001	0.67
Understanding other’s mind starting activities	9.53 ± 2.30 (9.21/9.84)	11.65 ± 1.99 (11.37/11.90)	11.24 ± 1.94 (10.97/11.50)	260.90 ( < 0.001)	< 0.001	< 0.001	0.56
Total Metacognition thinking skills	58.67 ± 9.93 (57.31/60.03)	69.53 ± 7.33 (68.52/70.54)	65.67 ± 7.96 (64.57/66.76)	904.39 ( < 0.001)	< 0.001	< 0.001	0.81

Abbreviations: F = one factor (time) repeated measure ANOVA; LB = Lower Bound; UB = Upper Bound

According to Table 4, there was a significant change over time in participants’ cooperative learning attitudes (F = 822.74; *p* < 0.001), indicating that the intervention was highly effective in enhancing cooperative learning attitudes across the study phases, with a large effect size ($\:{{\upeta\:}}_{p}^{2}$ = 0.80). The mean score of participants’ cooperative learning attitudes post-intervention (M (SD) = 49.05 (3.90)) showed a significant improvement compared to the pr-intervention score (M (SD) = 38.49 (3.46)) and this improvement was significant (*p* < 0,001). Additionally, the mean score of participants’ cooperative learning attitudes in follow up (M (SD) = 45.99 (3.62)) is higher than that of pre-training and this improvement was significant (*p* < 0,001). However, it was noted that the mean score at the follow-up stage decreased by 3.06 points compared to the post-intervention stage. Despite this slight decrease, the follow-up mean score still demonstrated a significant improvement from the pre-intervention stage, confirming the lasting positive impact of the intervention on cooperative learning attitudes.

**Table 4:** Changes in the mean score of participants cooperative learning attitude over time (*N* = 207)

Variable	Pre	post	3 months Follow-up	F (*p*)	Pairwise comparison (*p*)		$\:{{\upeta\:}}_{p}^{2}$
	Mean ± SD (LB/UB)	Mean ± SD (LB/UB)	Mean ± SD (LB/UB)		Pre-post	Pre-follow	
Cooperative learning attitude	38.49 ± 3.46 (38.02/38.96)	49.05 ± 3.90 (48.52/49.59)	45.99 ± 3.62 (45.49/46.49)	822.74 ( < 0.001)	< 0.001	< 0.001	0.80

Abbreviations: F = one factor (time) repeated measure ANOVA; LB = Lower Bound; UB = Upper Bound

## Discussion

This study aimed to evaluate the effects of implementing a problem-based learning approach training program for nursing students on their meta-cognitive thinking skills and cooperative learning attitude. The results demonstrated significant improvements in participants’ problem-based learning knowledge over time. These findings confirm the trainability of problem-based learning knowledge and its potential for sustained enhancement. The substantial increase in scores from pre-intervention to the post-intervention phase indicates the program’s immediate effectiveness in fostering this essential competency. This finding is consistent with previous research showing that structured educational programs can significantly enhance nurses’ knowledge [27, 28].

The problem-based learning approach training program also led to significant improvements in participants’ meta-cognitive thinking skills, with consistent gains across all assessed dimensions, including understanding one’s own mind, decentering and differentiation, mastery, and understanding others’ minds. The significant improvements in participants’ meta-cognitive thinking skills following the problem-based learning approach training program highlight its effectiveness in fostering higher-order cognitive abilities essential for reflective practice and critical decision-making in complex healthcare settings. This may be due to the structured and interactive nature of the problem-based learning approach, which encourages participants to actively engage with real-world scenarios, analyze complex problems, and reflect on their cognitive processes.

By promoting self-directed learning and collaborative problem-solving, the program likely enabled participants to develop a deeper understanding of their thought patterns (“understanding one’s own mind”) which aligned with point of view [29], recognize diverse perspectives (“decentering and differentiation”), and apply these insights effectively to achieve mastery in problem-solving and decision-making [30, 31]. Additionally, the program’s emphasis on active participation and critical reflection may have strengthened participants’ ability to empathize and understand others’ viewpoints (“understanding others’ minds”), further enhancing their meta-cognitive skills this notion was supported [32]. These improvements were sustained over time, reflecting the program’s comprehensive approach to developing higher-order cognitive skills. This aligning with previous studies of [17] that emphasized the role of metacognitive training in enhancing clinical decision-making. Among the individual dimensions, “decentering and differentiation” and “mastery” demonstrated the most substantial improvements, indicating that the intervention effectively equipped participants to approach challenges from multiple perspectives and achieve task competence. This may be due to the design of the problem-based learning approach, which inherently requires participants to analyze problems from various angles and consider alternative solutions, fostering “decentering and differentiation.” By encouraging collaboration and exposing participants to diverse viewpoints, the program likely enhanced their ability to step outside their own perspectives and evaluate situations more holistically. Although there was a slight decline in scores at follow-up, the maintained improvements indicate that participants retained a significant portion of the training’s benefits.

These findings are consistent with Paethrangsi et al. who highlighted the importance of continued practice to sustain gains in metacognitive skills [33]. So, the results of the study and the opinion of the reviewers confirmed the research hypothesis, which stated; Implementation of the problem-based learning approach program will enhance meta-cognitive thinking skills among nursing students at Faculty of Nursing, Port-Said University.

Additionally, the results demonstrated a substantial enhancement in cooperative learning attitudes following the intervention. The improvements observed suggest that participants quickly internalized the skills and strategies presented during the training. Perhaps, this may be related to the interactive and experiential nature of the training program, which likely engaged participants actively and made the learning process more impactful. These findings align with prior studies‏ of [28, 29] which emphasizing the effectiveness of targeted training programs in fostering cooperative learning attitudes, particularly those that incorporate interactive and experiential learning techniques. So, the results of the study and the opinion of the reviewers confirmed the research hypothesis which stated: implementation of the problem-based learning approach program will improve cooperative learning attitude among nursing students at Faculty of Nursing, Port-Said University.

### Limitations

This study has several limitations that warrant consideration. Firstly, the quasi-experimental design, while robust, does not include a control group, which may limit the ability to attribute observed changes directly to the intervention. Secondly, the participant group was limited to first and second-year nursing students from a single institution, which may reduce the generalizability of the findings to other nursing education contexts or to more advanced students. Another limitation is the potential bias introduced by self-reported measures, which can be influenced by participants’ desires to respond in a manner they perceive as favorable. Lastly, the timing of the intervention may have coincided with other academic pressures, despite efforts to schedule sessions on non-orientation days, which could affect the students’ engagement and the effectiveness of the program.

### Implications

The study offers several important implications for nursing education practice and policy. The positive outcomes associated with the problem-based learning program suggest that such approaches can significantly enhance meta-cognitive skills and cooperative learning attitudes among nursing students. These skills are critical for the practice of nursing, which requires ongoing learning and effective teamwork. Educational policymakers and curriculum developers should consider integrating problem-based learning strategies more comprehensively within nursing programs to foster essential skills in critical thinking, problem-solving, and collaboration. Moreover, this study supports the need for educational interventions that are carefully timed and integrated within nursing curricula to minimize conflict with other academic demands.

## Conclusions

The findings from this quasi-experimental study suggest that a problem-based learning program is a valuable educational tool for enhancing meta-cognitive thinking skills and cooperative learning attitudes among nursing students. The program’s structured approach, involving workshops and interactive activities, was effective in achieving these educational outcomes. However, further research is needed to explore these effects in a more diverse array of settings, include control groups to strengthen causal inferences, and assess the long-term impact of such educational interventions on professional practice. Additionally, future studies should consider the use of objective measures alongside self-reported data to provide a more comprehensive assessment of the intervention’s effectiveness.

## Electronic supplementary material

Below is the link to the electronic supplementary material.

Supplementary Material 1

## Data Availability

The datasets generated during and analyzed during the current study are not publicly available due to confidentiality agreements, but are available upon reasonable request from the corresponding author.
