# Supplementary material for: Effect of problem-based learning approach program on meta-cognitive thinking skills and cooperative learning attitude among nursing students: quasi experimental study
Source: BMC Nurs. 2025 Jul 10;24:899. doi: 10.1186/s12912-025-03485-z (PMC12247313; doi:10.1186/s12912-025-03485-z)
Supplement: Supplementary file 1 — Supplementary Material 1 [file 12912_2025_3485_MOESM1_ESM.docx]

Supplementary file 1. Semi-structured in-depth individual interview questions.

| **Category** | **Questions** |
| --- | --- |
| **Introduction** | Thank you for participating in this interview. The insights you provide today are invaluable to our study on medication errors in clinical settings. All information you share will be kept confidential and used solely for the purposes of this research. |
| **Main (Key)** | Can you describe your initial thoughts and feelings when you first encountered the PBL approach in our program? |
|  | What were your expectations regarding the PBL method at the beginning of the academic year? |
|  | Can you walk me through a typical PBL session? What activities do you engage in during these sessions? |
|  | How do these activities challenge or support your learning and thinking processes? |
|  | n what ways has the PBL approach influenced your ability to evaluate and regulate your own learning process? |
|  | Can you give an example of a situation where you had to apply your meta-cognitive skills to overcome a learning challenge? |
|  | Are there any elements of the PBL approach that you think could be improved? Please explain. |
| **Fixed** | Can you elaborate on that? |
|  | Can you give a specific example? |
|  | Are there any other instances or thoughts you would like to share on this topic? |
| **Additional** | Is there anything else you would like to add about your experiences in ? |
